# Primary Ewing sarcoma of the kidney: a rare entity with diagnostic challenges

**DOI:** 10.1093/jscr/rjae390

**Published:** 2024-06-03

**Authors:** Rihane El Mohtarim, Taha Yassine Aaboudech, Samia Sassi, Naji Rguieg, Amine Cherraqi, Ibrahima Diallo Dokal, Siham El Haddad, Nazik Allali, Latifa Chat, Laila Hessissen, Mounir Kisra, Lamiaa Rouas, Najat Lamalmi

**Affiliations:** Department of Pathology, Ibn Sina Teaching Hospital, Abderrahim Bouabid Avenue, Faculty of medecine and pharmacy, Rabat 12000, Morocco; Mohamed V University, Al Irfane, Rabat 12000, Morocco; Department of Pathology, Ibn Sina Teaching Hospital, Abderrahim Bouabid Avenue, Faculty of medecine and pharmacy, Rabat 12000, Morocco; Mohamed V University, Al Irfane, Rabat 12000, Morocco; Department of Pathology, Ibn Sina Teaching Hospital, Abderrahim Bouabid Avenue, Faculty of medecine and pharmacy, Rabat 12000, Morocco; Mohamed V University, Al Irfane, Rabat 12000, Morocco; Department of Pathology, Ibn Sina Teaching Hospital, Abderrahim Bouabid Avenue, Faculty of medecine and pharmacy, Rabat 12000, Morocco; Mohamed V University, Al Irfane, Rabat 12000, Morocco; Department of Radiology, Children’s Hospital, Faculty of medecine and pharmacy, Rabat 12000, Morocco; Department of Radiology, Children’s Hospital, Faculty of medecine and pharmacy, Rabat 12000, Morocco; Department of Radiology, Children’s Hospital, Faculty of medecine and pharmacy, Rabat 12000, Morocco; Mohamed V University, Al Irfane, Rabat 12000, Morocco; Department of Radiology, Children’s Hospital, Faculty of medecine and pharmacy, Rabat 12000, Morocco; Mohamed V University, Al Irfane, Rabat 12000, Morocco; Department of Radiology, Children’s Hospital, Faculty of medecine and pharmacy, Rabat 12000, Morocco; Mohamed V University, Al Irfane, Rabat 12000, Morocco; Pediatric Hematology-Oncology Service, Children’s Hospital, Faculty of medecine and pharmacy, Rabat 12000, Morocco; Mohamed V University, Al Irfane, Rabat 12000, Morocco; Department of Pediatric Surgery, Children’s Hospital, Faculty of medecine and pharmacy, Rabat 12000, Morocco; Department of Pathology, Ibn Sina Teaching Hospital, Abderrahim Bouabid Avenue, Faculty of medecine and pharmacy, Rabat 12000, Morocco; Mohamed V University, Al Irfane, Rabat 12000, Morocco; Department of Pathology, Ibn Sina Teaching Hospital, Abderrahim Bouabid Avenue, Faculty of medecine and pharmacy, Rabat 12000, Morocco; Mohamed V University, Al Irfane, Rabat 12000, Morocco

**Keywords:** renal Ewing sarcoma, histopathology, immunohistochemistry, molecular biology

## Abstract

Ewing sarcoma is a very rare tumour with aggressive behaviour and a poor prognosis. It tends to metastasize rapidly. Renal Ewing sarcoma is extremely rare, and only 48 cases have been reported in the literature. Herein, we report the case of a 14-year-old female presenting with a painful left flank swelling. Ultrasound and magnetic resonance imaging showed a large tumour invading the left kidney, heterogeneously enhanced after injection, associated with lymph nodes and peritoneal carcinomatosis. A thoraco-abdomino-pelvic computed tomography scan revealed pulmonary nodules and osteolytic lesions. A biopsy was performed, and histology, immunohistochemistry, and molecular studies confirmed the diagnosis of retroperitoneal Ewing sarcoma. Multi-agent chemotherapy followed by radical nephrectomy was performed, confirming the renal origin, and histology showed a post-therapeutical response. After a 1-year follow-up, there was no evidence of recurrence. We report this case to highlight the rarity of this entity and its challenging clinico-pathological diagnosis when presenting as a renal tumour.

## Introduction

Ewing sarcoma (EWS) is defined as a small round cell sarcoma showing gene fusions involving one member of the FET family of genes (usually EWSR1) and a member of the ETS family of transcription factors [1]. It occurs mostly in bones, while extraskeletal sites account for ~20%–30% of the cases [2]. Renal extraskeletal EWS is extremely rare, with 48 cases reported in the English literature to date [3]. It represents <1% of all renal tumours [4]. Definite diagnosis requires a pathological examination.

Herein, we report a new case of renal EWS in a 14-year-old female to highlight the rarity of this entity and its challenging clinico-pathological diagnosis when presenting as a renal tumour.

## Case report

A 14-year-old girl presented to the department of paediatric surgery with a 2-month history of abdominal pain and swelling of the flank and left hypochondrium, associated with generalized fatigue, anorexia, and weight loss. Physical examination of the patient revealed a left hypochondrial painful mass.

**Figure 1:**
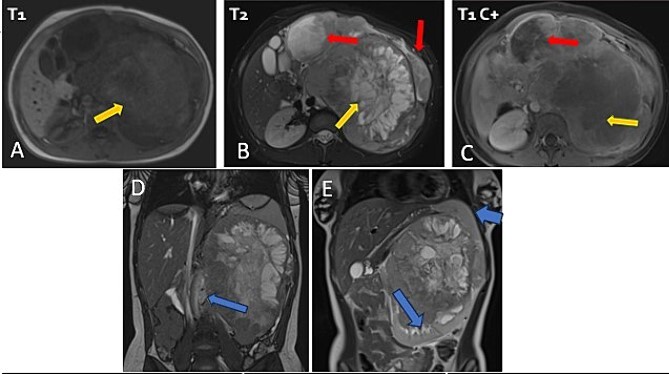
Abdominal MRI in axial T1 (A), T2 (B), and a T1 fat-saturated post contrast (Gadolinium) sequence (C), coronal T2 (D and E) showing a large retroperitoneal tumour process lateralized to the left, hypointense on T1-weighted, hyperintense, and heterogenous on T2-weighted images with central areas of necrosis and contrast-enhancing after Gadolinium administration. Lymph nodes and peritoneal carcinomatosis nodules are associated. Displacement of the vascular axes (D) and of the spleen of the left kidney responsible for left pelvicalyceal dilation (E).

**Figure 2:**
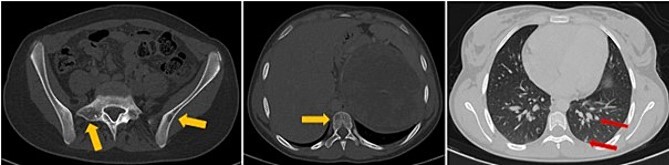
Axial thoraco-abdominal CT image on the bone window showing lytic bone lesions of the left iliac wing, sacrum, and vertebral bodies and on the pulmonary window showing pulmonary nodules.

The patient underwent sonography followed by magnetic resonance imaging (MRI), which showed a large lobulated retroperitoneal left tumour measuring 20 × 16 × 14 cm, which was hypointense on T1-weighted and heterogenous on T2-weighted images with necrotic components and contrast-enhancing after Gadolinium administration. This mass invaded the left kidney, causing homolateral pelvicalyceal dilatation (Fig. 1). Routine staging for metastasis showed: lymph nodes and peritoneal carcinomatosis nodules, pulmonary nodules revealed on a thoraco-abdomino-pelvic computed tomography (CT) scans, and secondary-looking osteolytic lesions (Fig. 2) confirmed by scintigraphy.

**Figure 3:**
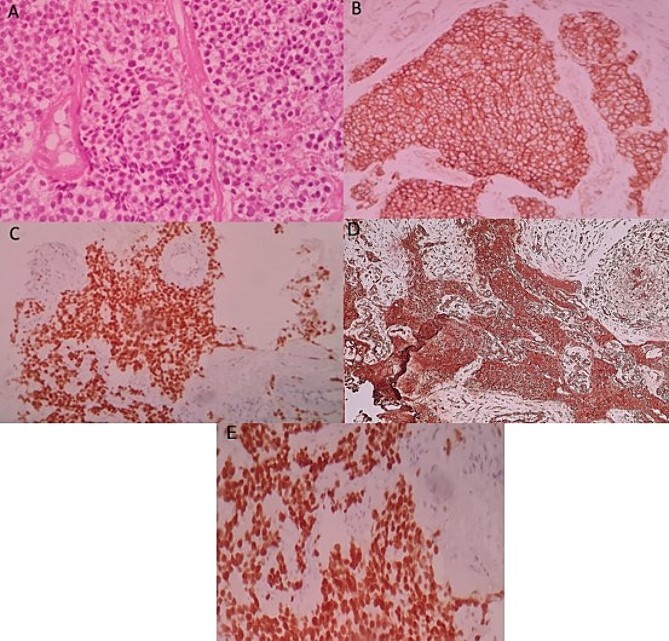
Histologic and immunohistochemical staining photomicrographs show sheets of small blue cells, scant cytoplasm with round or oval nuclei (A; H-E stain; original magnification, ×400), membranous expression of CD99 (B; CD99; original magnification, ×200), nuclear expression of NKX2-2 (C; original magnification, ×200), cytoplasmic expression of vimentine (D; original magnification, ×200), and nuclear expression of FLI1 (E; original magnification ×200), findings that are characteristic for EWS.

An ultrasound-guided biopsy was performed, and histopathology revealed medium-sized cells arranged in sheets and nests separated by fine fibro-vascular septae. The tumour cells had rounded to oval nuclei, finely granular chromatin, and pale-to-clear scanty cytoplasm (Fig. 3A). Immunohistochemistry showed positivity for: CD99, NKX2-2, FLI1, and vimentin (Fig. 3B–D). WT1, chromogranin, synaptophysin, CD45, CD3, CD20, desmin, and myogenin were negative. Therefore, nephroblastoma, neuroblastoma, lymphoma, and rhabdomyosarcoma were respectively eliminated. ERG was also negative.

A molecular study by fluorescent in situ hybridization confirmed the presence of an EWS-FLI1 fusion.

Based on radiological, pathological, and molecular findings, the definite diagnosis was retroperitoneal extra-skeletal EWS, although a primitive renal origin couldn’t be excluded.

The patient was referred to oncology department for treatment. The proposed regimen was two cures of VAC (vincristine, doxorubicin, and cyclophosphamide) and two cycles of alternating vincristine-doxorubicin-cyclophosphamide and ifosfamide-etoposide.

**Figure 4:**
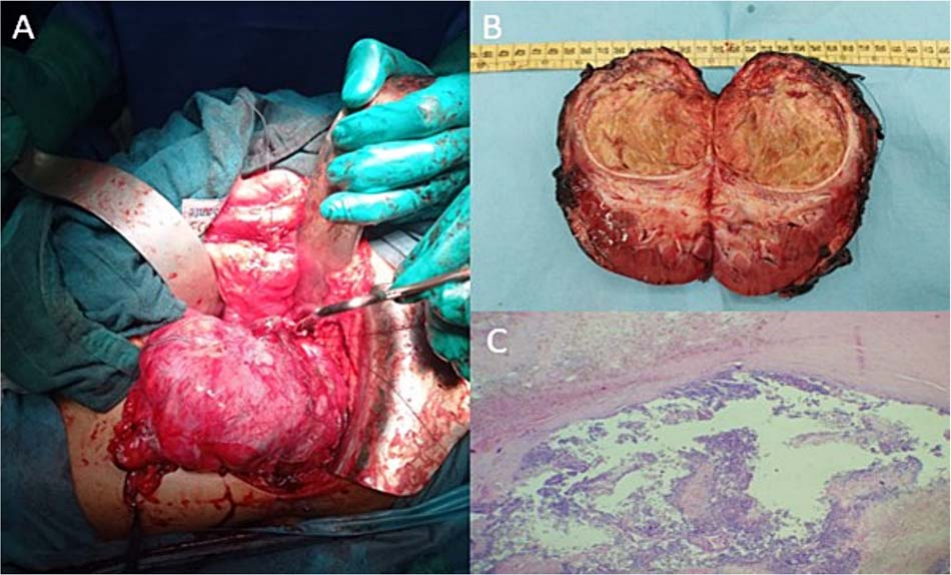
Intraoperative image showing the kidney and the tumour lesion (A) and the gross specimen of a nephroureterectomy (B) with histologic staining showing post-therapeutic changes of the tumour, consisting of fibrosis, necrosis, and inflammation (C; H-E stain; original magnification, ×200).

Post-therapeutical assessment showed a 94% response on the MRI. Therefore, the patient underwent radical nephrectomy, as intraoperative findings confirmed the primitive renal origin of the tumour (Fig. 4A). The specimen was sent to our department for pathological examination.

On gross, it was a well-circumscribed superior polar tumour measuring 7.3 × 7.8 × 5 cm with a whitish-yellow cut surface and largely necrotic appearance with an infiltrated capsule (Fig. 4B).

Microscopic findings showed extensive post-therapeutical changes consisting of fibrosis and necrosis (estimated at 95%) (Fig. 4C).

Scattered viable tumour cells were identical to those found in the previous biopsy. The tumour infiltrated the renal capsule. Hilar lymph nodes were negative for metastasis.

Immunohistochemistry confirmed the diagnosis of EWS.

The patient benefited from adjuvant chemotherapy (two cycles of VDC and IE). After a 1-year follow-up of the patient and maintenance treatment, clinical and imaging evidence demonstrated that there was no disease recurrence.

## Discussion

Renal EWS is a very rare tumour with aggressive behaviour and a poor prognosis. It tends to metastasize rapidly. The median age is 27 years old, with a slight male predominance [5]. The symptoms are not specific, including flank pain (84%), palpable mass (60%), and haematuria (38%) [6]. Radiological features are not specific and might be seen in many tumours, especially those seen in young adults, such as Wilms tumour, neuroblastoma, renal cell carcinoma, and lymphoma [7, 8].

The definitive diagnosis of renal EWS is based on pathological, immunohistochemical, and molecular testing.

On gross, EWS presents as greyish-white tumours with variable areas of haemorrhage and necrosis [9].

Microscopically, most cases are composed of uniform small round cells with round nuclei, finely stippled chromatin, inconspicuous nucleoli, scant clear or eosinophilic cytoplasm, and indistinct cytoplasmic membranes. Homer wright rosettes are common in renal ES, confirming their neuroectodermal differentiation [10].

Immunohistochemistry plays a pivotal role in the diagnosis of renal EWS, as new markers have improved the diagnostic accuracy, including NKX2.2.

NK2.2 is a protein that regulates the expression of genes involved in the neuroendocrine/glial differentiation pathway. NKX2.2 is a specific marker targeting the fusion protein EWS-FLI-1. It shows a high sensitivity of 93% and a specificity of 89% [11]. CD99 and FLI-1 are commonly used for EWS, though there is ongoing discussion about their precision [11].

The most frequent translocation in EWS is *t*(11,22) (q24;q12), which results in the *EWSR1-FLI1* fusion transcript seen in almost 85% of cases. The second most common one is *t*(21,22) (q22;q12), which results in *EWSR1-ERG* in ~10% of cases [1]. Fluorescent in situ hybridization (FISH) is the gold standard method with high sensitivity (92.3%) and specificity (100%) [12].

In sum, morphological, immunophenotypic, and molecular findings are mandatory to allow for the diagnosis of renal EWS/PNET and to rule out other small round blue cell tumours as well as common renal neoplams.

The differential diagnosis is broad, including rhabdomyosarcoma, neuroblastoma, lymphoma, and nephroblastoma [13]. That was the differential diagnosis we have ruled out in our case using specific markers for each pathology.

The prognosis is poor, with an overall 5-year disease-free survival of 45%–55%. No recurrence was reported for our patient after a follow-up period of 1 year.

There is no established standard of treatment for renal EWS because of its scarcity. The management of EWS typically encompasses surgical resection, coupled with adjuvant chemotherapy, with or without neoadjuvant chemotherapy, and radiotherapy [3].

Vincristine, doxorubicin, ifosfamide, etoposide, actinomycin D, and cyclophosphamide are the most effective chemotherapy drugs [14].

The use of insulin-like growth factor 1 receptor antibodies in molecularly targeted treatment has shown some promising potential in EWS [15].

## Conclusion

Renal EWS is a diagnostically challenging rare malignant tumour that should be kept in the differential diagnosis of small blue cell renal tumours. The radiological features are not specific, and diagnostic certainty is based on morphological, immunohistochemical, and molecular aspects. There is a need for a larger series to assess the prognosis and to define an effective therapy regimen.
